# Clinicopathological Parameters Predicting Nodal Metastasis in Head and Neck Squamous Cell Carcinoma

**DOI:** 10.7759/cureus.40744

**Published:** 2023-06-21

**Authors:** Atif A Hashmi, Rutaba Tola, Khushbakht Rashid, Abrahim H Ali, Tanim Dowlah, Umair Arshad Malik, Shamail Zia, Mubasshir Saleem, FNU Anjali, Muhammad Irfan

**Affiliations:** 1 Pathology, Liaquat National Hospital and Medical College, Karachi, PAK; 2 Internal Medicine, Karachi Medical and Dental College, Karachi, PAK; 3 Internal Medicine, Liaquat National Hospital and Medical College, Karachi, PAK; 4 Internal Medicine, Bangladesh Medical College, Dhaka, BGD; 5 Internal Medicine, Aga Khan University, Karachi, PAK; 6 Pathology, Jinnah Sindh Medical University, Karachi, PAK; 7 Dermatology, Dermcare Institute of Canada, Edmonton, CAN; 8 Internal Medicine, Sakhi Baba General Hospital, Sukkur, PAK; 9 Statistics, Liaquat National Hospital and Medical College, Karachi, PAK

**Keywords:** tumor differentiation, histological grade, tumor size, depth of invasion, clinicopathological parameters, nodal metastasis, squamous cell carcinoma, head and neck cancer

## Abstract

Introduction

Squamous cell carcinoma (SCC) is the most common type of malignancy of the head and neck region arising from the mucosal epithelium of the oral cavity and oropharynx. It is a multifactorial disease with a high rate of mortality. Lymph node metastasis is an important prognostic parameter associated with adverse prognosis. This study was conducted to establish a relationship between various clinicopathological characteristics and nodal metastasis in head and neck squamous cell carcinoma (HNSCC).

Methods

This retrospective study was conducted at Liaquat National Hospital, Karachi, Pakistan. A total of 306 biopsy-proven cases of HNSCC were included in the study. Clinical data, which included age, sex, and site of the lesion, were obtained from the clinical referral forms. Resections of the lesions were performed, and the specimens collected were sent to the laboratory for histological evaluation. The histological subtype, perineural invasion (PNI), depth of invasion (DOI), nodal metastasis, and extranodal extension were assessed, and the association of clinicopathological parameters with nodal metastasis was sought.

Results

The mean age at diagnosis was 50.26 ± 12.86 years with a female predominance (55.27%), and the mean tumor size was 3.37 ± 1.75 cm. The mean DOI was 1.08 ± 0.67 cm. The most common site of tumor was found to be the oral cavity (68.6%), followed by the tongue (24.2%). Keratinizing SCC (59.5%) was found to be the most prevalent histological subtype. At the time of diagnosis, the majority of the tumors were grade 2 (62.4%). PNI was present in 12.1% of the cases. Nodal metastasis was present in 44.8%, and extranodal extension was present in 17% of the cases. A significant association of nodal metastasis was noted with age, gender, tumor site, tumor size, and DOI. Male patients with HNSCC showed a higher frequency of nodal metastasis than female patients. Patients between the ages of 31 and 50 years with a tumor size of above 4 cm and a DOI of more than 1 cm had a higher frequency of nodal metastasis. Similarly, tumors arising in the oral cavity and the keratinizing subtype were more likely to possess nodal metastasis.

Conclusion

We found that HNSCCs were more prevalent among the female population, with the most common site being the oral cavity. Nodal metastasis was significantly associated with the keratinizing subtype of SCC, oral cavity location, male gender, and middle age group. Similarly, the tumor size and DOI were important predictors of nodal metastasis in HNSCC in our study.

## Introduction

Squamous cell carcinoma (SCC) is the most common type of malignancy of the head and neck region that develops from the mucosa of the oral cavity, pharynx, and larynx [1]. Head and neck squamous cell carcinoma (HNSCC) has been ranked as the sixth most common malignancy worldwide, with approximately 650,000 new cancer diagnoses and 330,000 deaths worldwide per year [2]. Over 90% of the oral and oropharyngeal malignancies are SCC [3]. It is a potentially fatal disease with a high mortality rate and a five-year survival rate of 50%, which decreases further in the presence of nodal metastasis to 20-36% [4,5]. The most common risk factors for developing HNSCC are tobacco use and alcohol consumption [6]. In certain Asian regions, betel nut chewing has been identified as the major and independent risk factor for developing HNSCC; in Western Europe and the United States, infection from human papilloma virus (HPV) has been identified as the major contributor to the increasing number of cases of oropharyngeal SCC [7].

Generally, males are found to have two to four times higher risk of developing HSNCC than females with a median age of 66 years in non-virally associated HNSCC and a median age of 53 years in virally associated HNSCC [1]. A high frequency of early cervical lymph node metastasis has been reported in HNSCC and is associated with poor outcome [8]. Lymph node metastasis in HNSCC is the strongest prognostic parameter and is associated with poor outcome [9]. The depth of invasion (DOI) is considered to be a useful prognostic factor and an important predictor of metastatic disease [5]. In 2007, the Union for International Cancer Control (UICC) and the American Joint Commission on Cancer (AJCC) made a few changes in the cancer staging of HNSCC, one of which is the addition of the DOI in the staging of oral cancers [10].

Data on the evaluation of clinicopathological characteristics of HNSCC and its association with lymph node metastasis are scarcely available in our population. The study aims to determine the relationship between various clinicopathological parameters and the presence of lymph node metastasis, which may help in identifying patients with HNSCC who are at a potentially higher risk of developing lymph node metastasis. Moreover, the radical neck dissection is associated with high morbidity, and there is a need to establish pathological parameters that are associated with nodal metastasis. Our study may help stratify patients in whom radical neck dissection can be avoided and selective neck dissection can be applied.

## Materials and methods

This is a retrospective cross-sectional study conducted at Liaquat National Hospital, Karachi, Pakistan, between February 2018 and January 2022. A total of 306 cases of HNSCC reported at our institute were included in the study. All biopsy-proven cases of HSCC were enrolled in the study. The clinicopathological data of the cases included in the study reported during the study period were retrieved from institutional archives. Clinical data, which included the age of the patient, gender, and tumor site, were obtained from clinical referral forms. Cases that were excluded from the study were those in which clinical data were missing. Cases that underwent neoadjuvant chemotherapy or radiation before surgical resection were also excluded from the study. In addition, cases with distant metastasis at the time of diagnosis were excluded. All the cases included in the study after the clinical examination and workup, including computed tomography (CT) scan and incisional biopsy, underwent surgery at our institute. The surgical margins of the specimens were assessed on frozen sections to ensure margin-free resection.

The resected specimens were sent to the laboratory in a 10% neutralized formalin-filled container for histological examination. After gross examination, the samples were kept in formalin for 24 hours at room temperature for fixation. Gross examination of the specimens was performed. Pathological parameters, such as tumor site, tumor configuration, and tumor size, were recorded, and representative sections were submitted from the tumor and margins. For tissue block preparation, the tissues were washed with water for an hour and were then dehydrated by treating the specimens with different concentrations of alcohol. Then, the samples were treated with xylene for three hours to clear off the alcohol from the tissue samples and were immersed in paraffin wax at 56°C. The formalin-fixed paraffin-embedded (FFPE) tissues were then sliced into 4-5 μm sections. The sliced sections were then transferred onto an L-lysine-treated slide; sequentially treated with xylene, alcohol, and water; and then stained with hematoxylin and eosin. The histological slides were studied by a senior histopathologist. The histological subtype, histological grade, perineural invasion (PNI), DOI, and nodal metastasis were assessed.

Data analysis

Data analysis was performed using IBM SPSS Statistics for Windows, Version 26.0 (Released 2019; IBM Corp., Armonk, New York, United States). The means of the patient’s age, size of the tumor, and DOI were calculated, along with the evaluation of the frequencies and percentages of all other clinicopathological variables. A p-value of <0.05 was considered significant. Chi-square and Fisher’s exact tests were applied to determine the association of various clinicopathological features with nodal metastasis.

## Results

A total of 306 cases of HNSCC were included in the study. Table 1 shows the clinicopathological features of the population in our study. The mean age was found to be 50.26 ± 12.86 years. We found that HNSCC was more prevalent in adults between the ages of 31 and 50 years (45.4%), whereas in 43.1% of cases, the patients were above 50 years of age. Most of the tumors (50.3%) ranged between 2.1 and 4.0 cm in size, with the mean size of the tumor being 3.37 ± 1.75 cm. Most tumors (50.7%) invaded 0.5-1.0 cm deep, with the mean DOI being 1.08 ± 0.67 cm. HNSCC was found to be more prevalent among females (55.2%) compared with males (44.8%). The most common site of occurrence was found to be the oral cavity (68.6%), with the second most common site being the tongue (24.2%). The most common histological subtype was found to be keratinizing SCC, present in 59.5% of cases, with the second most frequently diagnosed subtype being keratinizing with maturation (31%). The majority of tumors (62.4%) were found to be at grade 2 at the time of diagnosis. PNI was present in 12.1% of cases. Nodal metastasis was found in 44.8% of cases, and extranodal extension was found in 17% of cases.

**Table 1:** Clinicopathological parameters of the population under study SD: standard deviation

Clinicopathological parameters	Values
Age (years), Mean±SD	50.26±12.86
Age groups	
≤30 years, n (%)	35 (11.4)
31-50 years, n (%)	139 (45.4)
>50 years, n (%)	132 (43.1)
Tumor size (cm), Mean±SD	3.37±1.75
Tumor size groups	
≤2 cm, n (%)	64 (20.9)
2.1-4.0 cm, n (%)	154 (50.3)
>4 cm, n (%)	88 (28.8)
Depth of invasion (cm)	
Mean±SD	1.08±0.67
Depth of invasion groups	
<0.5 cm, n (%)	32 (10.5)
0.5-1.0 cm, n (%)	155 (50.7)
>1 cm, n (%)	119 (38.9)
Gender	
Male, n (%)	137 (44.8)
Female, n (%)	169 (55.2)
Sit e	
Oral cavity, n (%)	210 (68.6)
Lip, n (%)	5 (1.6)
Tongue, n (%)	74 (24.2)
Soft palate, n (%)	17 (5.6)
Histological subtypes	
Non-keratinizing, n (%)	29 (9.5)
Keratinizing, n (%)	182 (59.5)
Keratinizing with maturation, n (%)	95 (31)
Histological grade	
Grade 1, n (%)	87 (28.4)
Grade 2, n (%)	191 (62.4)
Grade 3, n (%)	28 (9.2)
Perineural invasion, n (%)	
Present	37 (12.1)
Absent	269 (87.9)
Nodal metastasis, n (%)	
Present	137 (44.8)
Absent	169 (55.2)
Extranodal extension	
Present, n (%)	52 (17)
Absent, n (%)	254 (83)

Table 2 demonstrates the association of the clinicopathological parameters with nodal metastasis in HNSCC. A statistically significant association was noted between gender, age, tumor size, DOI, tumor site, and histological subtype with nodal metastasis. We found that compared with females (34.3%), nodal metastasis was more common in males (65.7%). Moreover, adults between the ages of 31 and 50 years were more likely to have nodal metastasis. Nodal metastasis showed a positive association with tumor size, i.e., large tumors greater than 4 cm were more likely to show nodal metastasis (39.4%) compared with tumors of smaller size. Similarly, tumors with a DOI greater than 1 cm were more likely to have nodal metastasis (46%) compared with tumors of less than 0.5 cm and 0.5-1.0 cm (11.7% and 42.3%, respectively). HNSCC present in the oral cavity showed a higher frequency of nodal metastasis, present in 78.7% of the cases, compared with SCC involving the tongue, lip, and soft palate (18.3%, 2.4%, and 0.6% respectively). Keratinizing SCC (68.6%) was found to be more likely to present with nodal metastasis. No statistically significant association was established between nodal metastasis and PNI and histological grade.

**Table 2:** Association of clinicopathological parameters with nodal metastasis in head and neck squamous cell carcinoma *Chi-square test was applied, ** p-value significant as <0.05, **Fisher’s exact test was applied

Clinicopathological parameters	Values		p-value
	Nodal metastasis		
	Present	Absent	
Gender*			
Male, n (%)	90 (65.7)	140 (82.8)	0.001**
Female, n (%)	47 (34.3)	29 (17.2)	
Age groups*			
≤30 years, n (%)	12 (8.8)	23 (13.6)	<0.001**
31-50 years, n (%)	86 (62.8)	53 (31.4)	
>50 years, n (%)	39 (28.5)	93 (55)	
Tumor size groups*			
≤2 cm, n (%)	34 (24.8)	30 (17.8)	<0.001**
2.1-4.0 cm, n (%)	49 (35.8)	105 (62.1)	
>4 cm, n (%)	54 (39.4)	34 (20.1)	
Depth of invasion*			
<0.5 cm, n (%)	16 (11.7)	16 (9.5)	0.031**
0.5-1.0 cm, n (%)	59 (42.3)	97 (57.4)	
>1 cm, n (%)	63 (46)	56 (33.1)	
Site***			
Oral cavity, n (%)	133 (78.7)	77 (56.2)	<0.001**
Lip, n (%)	4 (2.4)	1 (0.7)	
Tongue, n (%)	31 (18.3)	43 (31.4)	
Soft palate, n (%)	1 (0.6)	16 (11.7)	
Histological subtypes*			
Non-keratinizing, n (%)	6 (4.4)	23 (13.6)	0.003**
Keratinizing, n (%)	94 (68.6)	89 (52.1)	
Keratinizing with maturation, n (%)	37(27)	58(34.3)	
Histological grade*			
Grade 1, n (%)	37 (27)	50 (29.6)	0.465
Grade 2, n (%)	90 (65.7)	101 (59.8)	
Grade 3, n (%)	10 (7.3)	18 (10.7)	
Perineural invasion*			
Present, n (%)	120 (87.6)	149 (88.2)	0.878
Absent, n (%)	17 (12.4)	20 (11.8)	

## Discussion

This study was conducted to evaluate the association of clinicopathological parameters with nodal metastasis in HNSCC. We found a positive association between patient gender, patient age, tumor size, DOI, tumor site, and histological subtype with the presence of nodal metastasis. We found that male patients with HNSCC were more likely to present with nodal metastasis than females. Moreover, patients between the ages of 31 and 50 years were more likely to have nodal metastasis compared with younger and older patients. Large tumors greater than 4 cm were more likely to have nodal metastasis. Similarly, we found that tumors with a DOI greater than 1 cm showed an association with nodal metastasis. Hence, we concluded that the deeper the tumor invaded, the greater the chances of nodal metastasis. SCC of the oral cavity showed a higher frequency of nodal metastasis, and the keratinizing subtype of SCC showed a higher association with nodal metastasis.

Goldson et al. [11] conducted a study on 644 cases of HNSCC to demonstrate the clinicopathological predictor of lymphatic metastasis. Unlike our findings, they found that tumors developing in the oropharynx and hypopharynx were more likely to show lymph node metastasis, whereas we found that tumors of the oral cavity showed a higher frequency of lymph node metastasis. They also found an association between tumor grade (poorly differentiated tumor) and lymphovascular invasion with positive lymph nodes, whereas our study failed to develop an association between tumor grade and lymph node metastasis. Unlike our study, they did not find an association between gender, tumor size, and PNI with lymph node metastasis.

Jangir et al. [12] conducted a study to predict the association between the DOI and the risk of nodal metastasis in oral cavity SCC. They found that tumors with a DOI greater than 5 mm were more likely to show nodal metastasis. Fukano et al. [13] conducted a study on 34 patients with tongue carcinomas and corroborated the finding that tumors with a DOI greater than 5 mm were more likely to be lymph node positive. Kane et al. [14] also demonstrated that a DOI greater than 5 mm is an important predictor of lymph node metastasis. Heft Neal et al. [15] conducted a study to predict the prevalence of nodal metastasis in salvage oropharyngectomy. They found an association between the female gender and the presence of nodal metastasis, whereas in our study, male patients with HNSCC were more likely to have nodal metastases. The other predictors of nodal disease in their study were the advanced stage of a primary disease and advanced/recurrent tumor (T) stage. Kurokawa et al. [16] conducted a study to evaluate the risk factors for late cervical node metastasis in 50 patients with tongue cancer. They concluded that tumors with sizes greater than 3 mm and DOIs greater than 4 mm were more likely to be associated with nodal metastasis.

Although we failed to establish a statistically significant association between PNI and tumor grade with nodal metastasis, several previous studies have demonstrated an association between tumor grade and PNI with positive lymph nodes [11,17].

Apart from histological features, the role of biomarkers in HNSCC has been studied. The markers that have been widely studied include programmed cell death ligand 1 (PD-L1), epidermal growth factor receptor (EGFR), and tumor suppressor gene products p16, p53, and p27 [18-25]. PD-L1 expression confers a promising response to targeted immunotherapy, whereas p53 and EGFR expressions are associated with poor prognostic parameters [20-23]. The risk factors and pathogenesis of HNSCC in Southeast Asia differ from those of Western countries, which are associated with a higher recurrence rate [18,24,25]. Therefore, there is a need to discover biomarkers that are associated with a dismal prognosis or can predict chemotherapy response. In our study, we did not evaluate prognostic biomarkers apart from histological parameters.

Limitations of the study

This study has a few limitations. First, the study was conducted in a single institute, and there was a limited sample size. Moreover, as this was a retrospective study, follow-up of the patients was not performed to determine the association of overall survival and disease-free survival with nodal metastasis. Similarly, risk factors were not evaluated, and therapeutic intervention studies were not conducted. Therefore, we propose that further prospective multicenter studies on HNSCC be conducted to better understand the relationship between various clinicopathological parameters and nodal metastasis. Moreover, we did not evaluate the association of various biomarkers with nodal metastasis.

## Conclusions

HNSCC is more prevalent among adults between the ages of 31 and 50 years in our region, with female predominance. We found a significant association among age, gender, tumor size, DOI, tumor site, and tumor type with nodal metastasis in HNSCC. Female patients between the ages of 31 and 50 years with oral cavity tumors and keratinizing SCC were more likely to have nodal metastasis. Moreover, the risk of nodal metastasis increases with increasing tumor size and DOI. This study emphasizes the key pathological predictors of nodal metastasis in HNSCC and may help stratify patients in whom neck dissection can be avoided.
